# Co-occurrence of Spondyloepiphyseal Dysplasia and X-Linked Hypophosphatemia in a Three-Generation Chinese Family

**DOI:** 10.1007/s00223-023-01104-0

**Published:** 2023-06-06

**Authors:** Jian Ma, Ye Zhang, Xiaoxiao Ding, Zhijiang Liang, Chaoxiang Yang, Zhi Deng, Hui He, Zhihong Guan, Chunhua Zeng, Yunting Lin, Xianqiong Luo

**Affiliations:** 1 Translational Medicine Center, Guangdong Women and Children Hospital, Guangzhou, 511442 China; 2 Department of Pediatric Endocrinology and Inherited Metabolic Diseases, Guangdong Women and Children Hospital, Guangzhou, 511442 China; 3 Department of Child Health, Shenzhen Maternity & Child Healthcare Hospital, Shenzhen, 518028 China; 4 Department of Public Health, Guangdong Women and Children Hospital, Guangzhou, 511442 China; 5 Department of Radiology, Guangdong Women and Children Hospital, Guangzhou, 511442 China; 6 Department of Genetics and Endocrinology, Guangzhou Women and Children’s Medical Center, Guangzhou Medical University, Guangdong Provincial Clinical Research Center for Child Health, Guangzhou, 510623 China

**Keywords:** Genetic skeletal disorders, *COL2A1* gene, Spondyloepiphyseal dysplasia, *PHEX* gene, X-linked hypophosphatemia

## Abstract

Rare genetic skeletal disorders (GSDs) remain the major problem in orthopedics and result in significant morbidity in patients, but the causes are highly diverse. Precise molecular diagnosis will benefit management and genetic counseling. This study aims to share the diagnostic experience on a three-generation Chinese family with co-occurrence of spondyloepiphyseal dysplasia (SED) and X-linked hypophosphatemia (XLH), and evaluate the therapeutic effects of two third-generation siblings. The proband, his younger brother, and mother presented with short stature, skeletal problems, and hypophosphatemia. His father, paternal grandfather, and aunt also manifested short stature and skeletal deformities. Whole exome sequencing (WES) of proband–brother–parents initially only found the proband and his younger brother had a pathogenic c.2833G > A(p.G945S) variant in the *COL2A1* gene inherited from their father. Re-analysis of WES uncovered the proband and his younger brother also harbored a pathogenic ex.12 del variant in the *PHEX* gene transmitted from their mother. Sanger sequencing, agarose gel electrophoresis, and quantitative polymerase chain reaction proved these results. The proband and his younger brother were confirmed to have a paternally inherited SED and a maternally inherited XLH. During a 2.8-year follow-up, these two siblings remained short stature and hypophosphatemia, but their radiographic signs and serum bone alkaline phosphatase levels were improved with treatment of oral phosphate and calcitriol. Our study presents the first report of co-occurrence of SED and XLH, shows the possibility that two different rare GSDs co-exist in a single patient, and alerts clinicians and geneticists to be cautious about this condition. Our study also suggests that next-generation sequencing has limit in detecting exon-level large deletions.

## Introduction

Genetic skeletal disorders (GSDs), also known as genetic skeletal dysplasias, comprise a large set of genetic disorders affecting the skeletal development and growth with an overall prevalence of 1/5000-1/3000 births [1–4]. The typical manifestations of GSDs include short stature, skeletal deformities, osteoporosis, osteopenia, bone pain, osteoarthritis, and fracture. Rare GSDs remain the major problem in orthopedics and result in significant morbidity in patients. According to the newest nosology in 2023, 771 different conditions and 552 different causative genes have been recognized in GSDs [5].

A definite diagnosis is critical to predict prognosis, guide management, and provide counseling for patients. However, clinical complexity, genetic heterogeneity, and phenotypic similarity among GSDs challenge the diagnosis. For example, *COL2A1* gene defects could result in a spectrum of phenotypes ranging from milder premature arthritis to more severe bone dysplasias to perinatally death [6–8]. Another example is that spondyloepiphyseal dysplasia (SED) could be arisen from variants in a range of genes, including *COL2A1*, *TRPV4*, *ACAN*, *TRAPPC2*, *CHST3*, *NMNAT1*, *MBTPS1*, *COMP*, *CCN6*, *MIR140*, and *RNU4ATAC*, which are documented in Online Mendelian Inheritance in Man (OMIM, https://www.omim.org/). Only a few of GSDs having distinct characteristics could achieve an accurate diagnosis [6], such as X-linked hypophosphatemia (XLH) caused by loss-of-function variants in the *PHEX* gene.

Distinguished from classic Sanger sequencing targeting a specific gene, next-generation sequencing (NGS), including whole genome sequencing (WGS), whole exome sequencing (WES), and target region sequencing (TRS), enables rapid screening of multiple candidate genes [9], which could contribute to the precise molecular diagnosis of GSDs, especially when the clinical diagnosis is blurred. However, NGS is weak to identify some special variants, such as mosaicism [10, 11], as well as large deletions covering more than one exon [12]. A subsequent confirmatory Sanger sequencing or a supplementary quantitative polymerase chain reaction (qPCR) or multiplex ligation-dependent probe amplification (MLPA) assay will help to improve detection efficiency and sensitivity.

In this study, we presented a three-generation Chinese family with co-occurrence of SED and XLH, and analyzed their clinical, radiological, biochemical, and genetic findings. Two third-generation siblings with skeletal dysplasia and hypophosphatemia harbored both *COL2A1* and *PHEX* pathogenic variants which co-segregated with phenotypes across the paternal and maternal pedigrees. We also reviewed the 2.8-year follow-up of these two siblings and evaluated the therapeutic effects.

## Materials and Methods

### Patients

**Fig. 1:**
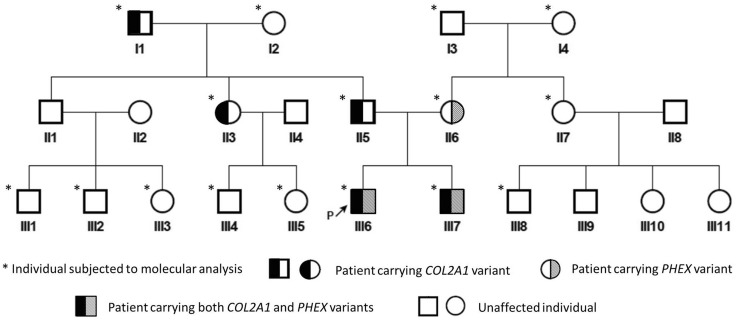
The pedigree of the three-generation family with GSDs

The three-generation family with GSDs (Fig. 1) was enrolled in Guangdong Women and Children Hospital and Guangzhou Women and Children’s Medical Center. All subjects are Chinese and are of Han ethnicity. All couples are non-consanguineous.

The clinical information was collected and evaluated by clinicians. Physical examinations were performed by specialist physicians, X-ray examinations were performed by radiologists, ultrasonography was performed by sonologists, and family history was inquired by genetic counselors. Biochemical and hormonal parameters of blood samples were detected in hospital’s clinical laboratory center and endocrinology and metabolism laboratory, respectively.

### Molecular Genetic Analysis

Six members with skeletal abnormalities and ten unaffected individuals were subjected to molecular genetic analysis (Fig. 1). Genomic DNA (gDNA) was extracted from peripheral blood samples using DNeasy Blood and Tissue Kit (QIAGEN, Hilden, Germany). WES was performed as previously described [13, 14]. To validate the *COL2A1* variant identified by WES, the classic Sanger sequencing of the PCR products targeting the exon 42 of the *COL2A1* gene (NG_008072.1, NM_001844.5) was carried out. For large deletion covering the exon 12 of the *PHEX* gene (NG_007563.3, NM_000444.6), an agarose gel electrophoresis of the PCR products and a qPCR of gDNA were conducted as previously described [15].

### Treatment and Follow-up

After diagnosis of hypophosphatemia, the proband and his brother were treated with oral phosphate (Guangzhou Women and Children’s Medical Center, Guangzhou, China) in combination with calcitriol [Roche Pharma (Schweiz) Ltd., Shanghai, China] supplementation. Clinical follow-up was performed at an interval of 2 weeks to 4 months. The height, weight, and related biochemical parameters were measured at an appropriate interval.

## Results

### Clinical, Radiological, and Biochemical Features

**Fig. 2:**
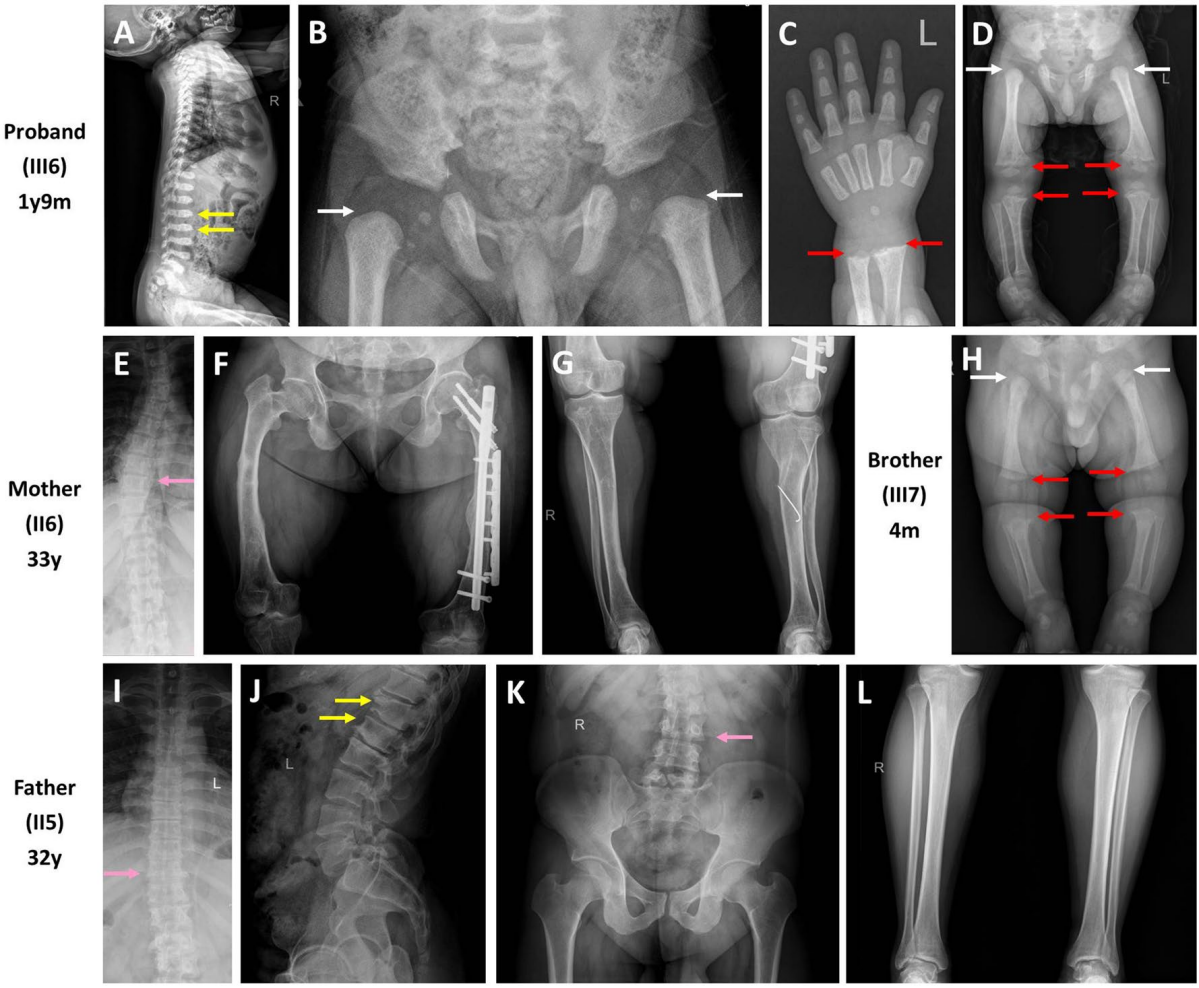
The skeletal X-ray images of the four core patients in the enrolled family. **A–D**. The skeletal X-ray images of the proband (III6). **E–G**. The skeletal X-ray images of the mother (II6). **H**. The skeletal X-ray images of the brother (III7). **I–L**. The skeletal X-ray images of the father (II5). The yellow arrow indicates flattened and transverse bottles-like vertebral body, the white arrow indicates small epiphyses of the femoral heads, the red arrow indicates widened and brush-like metaphyses, and the pink arrow indicates scoliosis

The proband (III6) was the first child of his symptomatic parents with a birth length of 51.0 cm [0.3 standard deviation (SD)] and a birth weight of 3.9 kg (1.4 SD) at full-term gestation (Fig. 1) [16]. He was found to have shortened limbs after birth. He first visited our clinic at 1 year and 9 months of age because of failing to thrive, short stature (73.0 cm, − 4.0 SD), and genu varum (Table 1). His spine X-ray presented flattened and transverse bottles-like vertebral body (Fig. 2A), pelvis X-ray manifested small epiphyses of the femoral heads and coxa vara (Fig. 2B), and limb X-ray showed rachitic signs characterized by a general decrease in bone density, widened and brush-like metaphyses of the distal radius, ulna and femurs, and the proximal tibia and fibula, small epiphyses of the femoral heads, and shortened long bones (Fig. 2C and D). Laboratory examinations at 1 year and 11 months of age revealed decreased serum phosphate level, elevated serum bone alkaline phosphatase (BALP) activity, along with normal serum calcium, 25(OH) vitamin D, and parathyroid hormone (PTH) levels. The tubular reabsorption of phosphate (TRP) was mildly impaired with a value of 77.0%. The level of fibroblast growth factor 23 (FGF23) was significantly elevated to 254.3 pg/mL (Table 2) [17]. A diagnosis of hypophosphatemia was then made based on these findings.

**Table 1:** Clinical, radiological, and molecular characteristics of six patients in this family at diagnosis

Patient	Gender	Age	Height (cm)	Variant	Clinical symptoms	Radiological findings
I1	M	56y	162.0 (− 1.8 SD)	*COL2A1*: c.2833G > A(p.Gly945Ser) het	Osteonecrosis of the femoral heads, leg pain	NA
II3	F	31y	140.0 (− 3.8 SD)	*COL2A1*: c.2833G > A(p.Gly945Ser) het	Scoliosis, shortened limbs, groin and back pain, difficulty in walking	NA
II5	M	32y	153.0 (− 3.2 SD)	*COL2A1*: c.2833G > A(p.Gly945Ser) het	Scoliosis, shortened limbs, groin and back pain	Scoliosis, abnormal vertebral morphology
II6	F	33y	133.0 (− 5.1 SD)	*PHEX*: ex.12 del het	Scoliosis, genu varum	Scoliosis, decreased bone density
III6	M	1y11m	73.2 (− 4.3 SD)	*COL2A1*: c.2833G > A(p.Gly945Ser) het *PHEX*: ex.12 del hemi	Shortened limbs, genu varum, coxa vara	Abnormal vertebral morphology, small epiphyses of the femoral heads, rachitic signs, shortened long bones
III7	M	4m	56.5 (− 3.6 SD)	*COL2A1*: c.2833G > A(p.Gly945Ser) het *PHEX*: ex.12 del hemi	Shortened limbs, polydactyly of the left thumb	Rachitic signs, small epiphyses of the femoral heads, shortened long bones

*M* Male, *F* Female, *y* year, *m* month, *SD* standard deviation, *del* deletion, *het* heterozygous, *hemi* hemizygous, *NA* not available

The height is evaluated in terms of the standardized growth charts for Chinese children and adolescents from birth to 18 years [15]

**Table 2:** Biochemical parameters of five patients in this family at diagnosis

Patient	Reference range for adults	II3	II5	II6	Reference range for children	III6	III7
Age	> 18y	31y	32y	33y	0–6y	1y11m	4 m
Serum phosphate (mmol/L)	0.81–1.55	1.10	1.10	0.76↓	1.29–2.26	0.79↓	0.91↓
Serum calcium (mmol/L)	1.95–2.80	2.29	2.50	2.45	1.95–2.80	2.43	2.62
Serum BALP (ug/L)	3.70–20.90 for males 2.90–14.50 for females	–	11.74	35.13↑	39.20–159.50	166.74↑	268.51↑
Serum 25(OH) vitamin D (nmol/L)	75.0–250.0	18.4↓	82.7	40.1↓	75.0–250.0	125.3	111.4
Serum PTH (pmol/L)	1.30–6.80	93.8↑	3.16	6.19	1.30–6.80	5.93	1.22↓
TRP (%)	85.0–95.0	–	–	–	85.0–95.0	77.0↓	83.4↓
FGF23 (pg/mL)	9.0–50.4	–	28.0	58.8↑	9.0–50.4	254.3↑	187.5↑

*BALP* bone alkaline phosphatase, *PTH* parathyroid hormone, *TRP* tubular reabsorption of phosphate, which is calculated according to the formula of [1-(urine phosphate × serum creatinine)/(serum phosphate × urine creatinine)] × 100%, *FGF23* fibroblast growth factor 23, *y* year, *m* month, *-* not done

The mother (II6) had severe skeletal deformities and had undergone surgery during childhood. She currently presented with short stature, scoliosis, and genu varum (Table 1). Scoliosis and diffusely decreased bone density were revealed by X-ray (Fig. 2E–G), while decreased serum phosphate level, and increased BALP and FGF23 levels were detected in the mother (Table 2), supporting the same diagnosis of hypophosphatemia as her son. The mother’s parents (I3 and I4) and sister (II7) were all healthy (Fig. 1).

The proband’s 4-month-old younger brother (III7) further received screening test. He had mild delay of height development, but no obvious deformity was noticed in his spine and lower extremities. He was born at 36 weeks of gestation with a birth length of 44.0 cm (− 3.7 SD) and a birth weight of 2.4 kg (− 2.6 SD), and was found with shortened limbs and syndactyly of the left thumb (Table 1). He shared similar short stature, radiological rachitic signs, and biochemical phenotypes with the proband (Tables 1, 2, and Fig. 2H), inferring an identical disease to his brother.

Additionally, the proband’s father (II5), paternal grandfather (I1), and aunt (II3) also had short stature and skeletal deformities with variable degrees (Table 1), whereas other paternal family members were unaffected (Fig. 1), indicating a family history of an autosomal dominant GSD. X-ray revealed normal bone density, but scoliosis along with flattened and transverse bottles-like vertebral body in the spine of the father (Fig. 2I–K), no obvious abnormality found in his pelvis and lower extremities (Fig. 2K and L). Elevated PTH level was shown in the aunt, whereas the serum biochemical indexes of the father were almost normal (Table 2).

### Genetic Findings

As the diagnosis of hypophosphatemia was made in the two siblings and their mother, a dominant inheritance trait was suggested. Besides, an autosomal dominant GSD was also indicated in the paternal pedigree. It seems that two different disorders co-existed in this three-generation family. To identify the phenotype-producing variants of skeletal problems in this family, the four core family members (proband–brother–parents) were included in WES with a focus on the causative genes of GSDs.

**Fig. 3:**
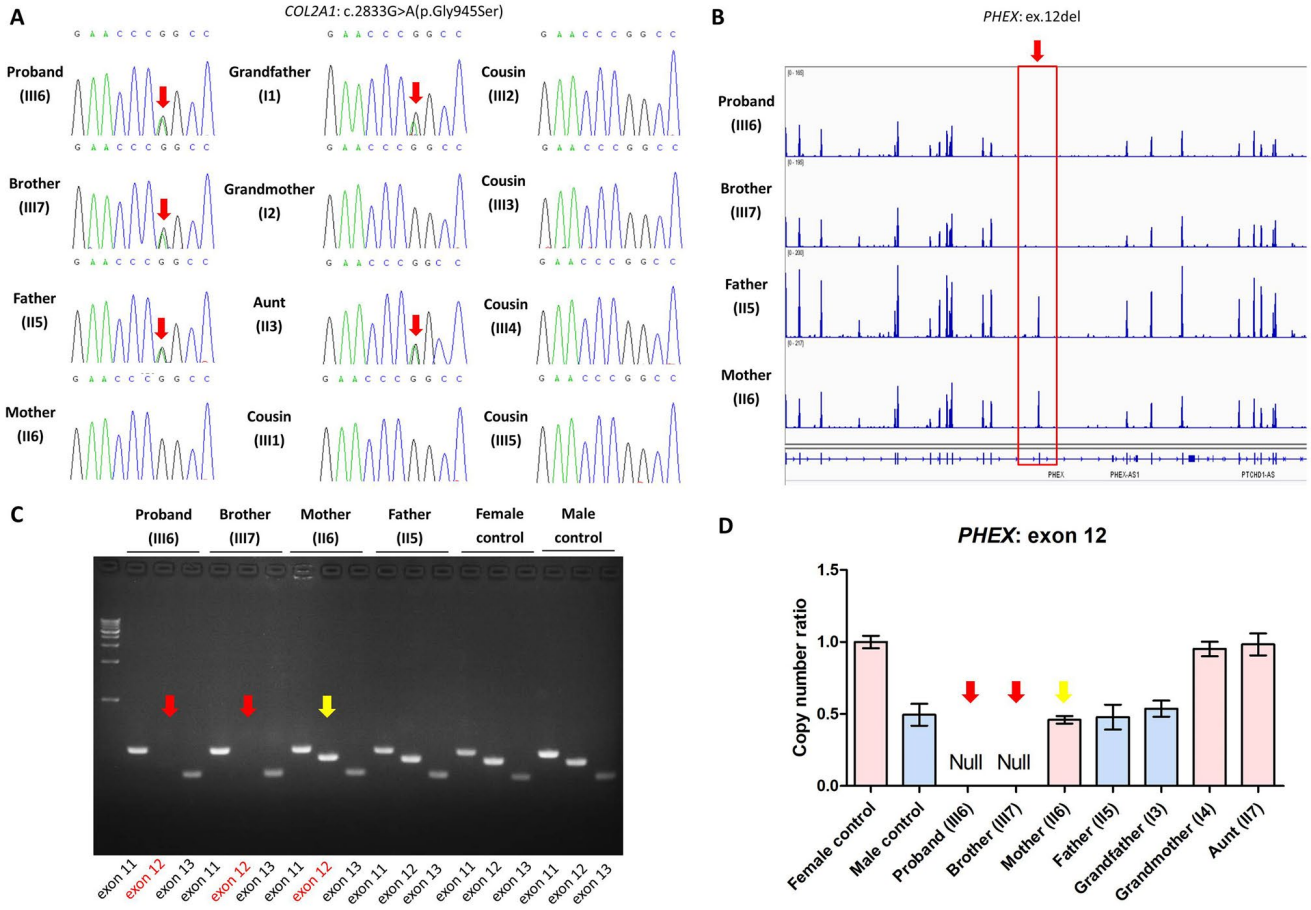
The molecular findings of the enrolled family. **A**. The Sanger sequencing diagrams. The red arrow indicates the *COL2A1* c.2833G > A(p.G945S) variant. **B**. The electronic read diagrams of WES mapped to the *PHEX* gene. The red frame and arrow indicates the exon 12 in the *PHEX* gene. Since the *PHEX* gene is located on the X chromosome, the copy number is twice in females than males. The absence of a peak in the proband and brother represents hemizygous ex.12 del, while the mother with a similar peak height with the father represents heterozygous ex.12 del. **C**. The agarose gel electrophoresis diagrams. The red arrow indicates the absence of band caused by hemizygous ex.12 del, whereas the yellow arrow shows the existence of a band as the product heterozygous ex.12 del. **D**. The qPCR results. The normal copy number ratio in comparison with control gene is 1.0 in females showed as red columns and 0.5 in males showed as blue columns. The red arrow indicates hemizygous ex.12 del in the proband and brother, while the yellow arrow indicates heterozygous ex.12 del in the mother

A heterozygous c.2833G > A(p.G945S) missense variant in the *COL2A1* gene, which is known to be responsible for SED or Spondylometaphyseal dysplasia (SMED) [8, 18], was found in the proband, and his father and brother. Sanger sequencing subsequently revealed the presence of this *COL2A1* variant in the affected grandfather, aunt, father, proband, and brother, whereas the absence in other healthy subjects, which was co-segregated with phenotypes across the paternal pedigree (Fig. 3A). Given the findings of normal height or short-limbed short stature, platyspondyly, bone pain, small epiphyses, and coxa vara among the paternal family, a diagnosis of SED was made.

However, to our surprise, no suspicious variant was called by automatic bioinformatic analysis of WES in the mother as well as the proband and brother as the reason of hypophosphatemia, though a diagnosis was suggested by biochemical parameters (Table 2). In hypophosphatemia, XLH is the most frequent form accounting for over 80% of familial cases and over 70% of sporadic cases [15, 19]. Our previous study had shown that 12.3% XLH patients (8/65) were caused by gross deletions in the *PHEX* gene covering certain entire exons and flanking sequences [15], which was further supported by our in-house study with an increased cohort size (12.4%, 16/129).

To further determine the underlying genetic basis of hypophosphatemia in this family, the original electronic read data of WES were re-analyzed to investigate if there were gross deletions in the *PHEX* gene. As expected, a hemizygous deletion of exon 12 in the *PHEX* gene was observed in the proband and his brother, while their mother exhibited a heterozygous deletion (Fig. 3B). Agarose gel electrophoresis as well as qPCR-based copy number analysis confirmed this deletion in the affected mother and her sons, whereas the unaffected grandfather (I3), grandmother (I4), and aunt (II7) from the maternal pedigree did not carry it (Fig. 3C and D), indicating a de novo status in the mother. As the *PHEX* ex.12 del is a known pathogenic variant leading to an in-frame deletion [15, 20, 21], the proband, and his brother and mother were definitely diagnosed as XLH.

Thus, both the proband and his younger brother harbored a c.2833G > A(p.G945S) variant in the *COL2A1* gene and an ex.12 del in the *PHEX* gene, and were suffered from a paternally inherited SED and a maternally inherited XLH.

### Treatment and Follow-up

**Fig. 4:**
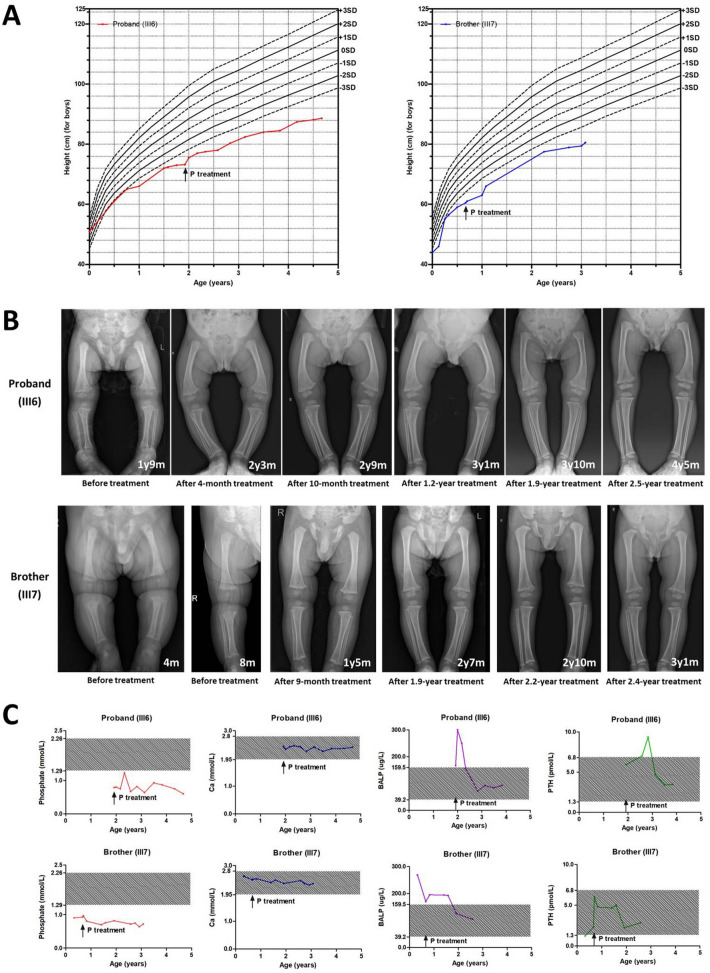
The therapeutic effects of conventional XLH treatment in the proband and his younger brother. **A**. The growth curves. **B**. The skeletal X-ray images. **C**. The biochemical parameters

These two siblings kept regular clinic visits and had a 2.8-year follow-up. After the diagnosis of hypophosphatemia at 1 year and 11 months of age, the proband was supplemented with oral phosphate and calcitriol in a dose of 60 mg/kg/day, 6 times/day and 50 ng/kg/day, 2 times/day, respectively. Until 4 years and 8 months of age with a 2.8-year treatment, his height remained below − 3 SD and increasingly deviated from the normal range (Fig. 4A). His lower limb deformities were alleviated significantly (Fig. 4B). Additionally, his serum phosphate remained below the lower limit of normal range, but normalization achieved in his BALP level. His serum calcium and PTH remained normal (Fig. 4C).

The younger brother was clinically diagnosed as hypophosphatemia at 4 months of age, but started treatment with oral phosphate and calcitriol at 8 months of age. He manifested similar changes in growth curve, radiographic signs, and biochemical indexes as the proband (Fig. 4).

## Discussion

It is unusual that two different rare disorders co-exist in a single individual. Consanguineous marriages may increase the co-occurrence risk of rare inherited conditions, especially autosomal recessive disorders. The increasing probability to recognize the coincidence of two distinct inherited disorders achieves when they affect two different systems with diverse manifestations, whereas the involvement of the same system with phenotypic overlap may pose a differential diagnostic challenge. The use of genetic approaches, especially NGS, as well as the notice of valuable clues in clinically will prompt the resolution of this complex condition [22–28].

In this study, we shared the diagnostic process of two siblings with co-occurrence of SED and XLH from a three-generation family. As shown in Fig. 1, skeletal problems were presented in both the paternally and maternally pedigree. WES and confirmatory Sanger sequencing only identified a SED-causative variant in the *COL2A1* gene among the paternally pedigree initially. However, the *COL2A1* variant was not responsible for the hypophosphatemia of the two siblings, and their mother also suffered from hypophosphatemia. To find the true mechanism causing hypophosphatemia, we engaged a re-analysis of WES and uncovered an exon deletion in the *PHEX* gene. To the best of our knowledge, this is the first report of co-occurrence of SED and XLH caused by *COL2A1* and *PHEX* pathogenic variants in a single individual.

Actually, as both the *COL2A1* and *PHEX* gene play a role in the development of skeleton and are causative genes of GSDs with overlapped phenotypes of short stature, skeletal deformities, and bone pain, the diagnosis of XLH might be concealed. Fortunately, the well-delineated biochemical findings and the symptoms of the mother alert us to check the raw data of WES and avoid the missing of XLH diagnosis, which suggests that detailed family history investigation, necessary examinations, and sufficient attention are critical in medical evaluation.

NGS is a powerful sequencing approach widely used in the identification of disease-causing variants. However, the technical limitations and bioinformatic analysis pipelines limit NGS to identify all variants and may conceal “traps,” such as mosaicism [10, 11], as well as large deletions covering more than one exon [12]. In this study, the deletion of exon 12 in the *PHEX* gene was missed by automatic bioinformatic analysis initially, whereas retrieved by manual re-analysis of raw data, indicating that our bioinformatic analysis pipeline should be modified to better estimate the exon-level copy number variants.

The *PHEX* gene structure is unstable and sensitive to mutagenesis, and high spontaneous rate of de novo mutation is observed in XLH [15, 29]. In our previous study, 63.1% XLH patients (41/65) had a de novo *PHEX* variant, and postzygotic spontaneous mutation led to somatic mosaicism in 6.2% XLH patients (4/65) [15]. Moreover, large deletions covering more than one exon possess a great proportion in *PHEX* variants [15, 30]. In this study, the *PHEX* ex.12 del was present in the mother with a heterozygous status but absent in her parents, suggesting a de novo mutagenesis proceeded during or before the period of zygogenesis.

During the 2.8 years of follow-up, the conventional treatment of XLH consisted of oral phosphate and calcitriol supplementation was conducted in these two siblings. Although high dose of oral phosphate (60 mg/kg/day) was administered, their serum phosphate was still below the lower limit of normal range. With conventional therapy in XLH, the goal is to improve growth, normalize ALP, and avoid secondary hyperparathyroidism and nephrocalcinosis, while serum phosphate often could not reach the normal range [31]. Our cases showed improved radiographic alternations and normal levels of BALP and PTH, which fits the therapy goal of XLH. However, the growth curves after the conventional treatment of XLH were not as expected in our patients with increasing deviation from the normal range. This condition may be resulted from the synergistic effect of SED.

A newly approved treatment of burosumab subcutaneous injection could achieve normal serum phosphate levels and significant improvement of growth in few weeks [32–34]. Transition from conventional therapy to burosumab therapy is going in our cases. We will follow the outcomes of this new treatment in these two siblings in the future.

In addition, the two siblings and their grandfather, aunt, and father from the paternal pedigree suffered from SED but with variable severities, showing an intra-familial phenotypic heterogeneity of *COL2A1*-related SED. Surgical interventions might be needed in SED [35–37].

In summary, our study adds the first report of co-occurrence of SED and XLH caused by pathogenic *COL2A1* and *PHEX* variants in two siblings from a three-generation family, describes their phenotypic and genotypic features, shares experience on the diagnostic process, and presents the follow-up and therapeutic effects. Our cases alert clinicians and geneticists to be cautious about the coincidence of two different rare disorders in a single patient, especially when they affect the same system. Our report also shows the limitation of NGS in detecting exon-level large deletions and the need to modify the bioinformatic analysis pipeline.

## Data Availability

The data that support the findings of this study are available from the corresponding author upon reasonable request.
